# Be prepared and do the best you can: a focus group study with staff on the care environment at Swedish secure youth homes

**DOI:** 10.1080/17482631.2023.2168234

**Published:** 2023-02-02

**Authors:** Kajsa Nolbeck, Sepideh Olausson, Göran Lindahl, Charlotta Thodelius, Helle Wijk

**Affiliations:** a Institute of Social Work, University of Gothenburg, Gothenburg, Sweden; b Institute of Health and Care Sciences, Sahlgrenska Academy, University of Gothenburg, Gothenburg, Sweden; c Sahlgrenska University Hospital, Gothenburg, Sweden; d Department of Architecture and Civil Engineering, Division of Building Design, Chalmers University of Technology, and Centre for Healthcare Architecture, Gothenburg, Sweden; e Faculty of Police Work, University of Borås, Borås, Sweden

**Keywords:** Institutional youth care, sociomateriality, focus group discussions, thematic analysis, care environment, Erving Goffman

## Abstract

This study focuses on the care environment of secure youth homes run by the Swedish National Board of Institutional Care, where youths up to 21 years old are placed according to law due to extensive care needs or sentences.

**Purpose:**

This study examined staff members’ experiences of the institutional care environment within secure youth homes.

**Methods:**

Data were collected through three focus group discussions with 17 staff members at two secure youth homes. Subsequently, a thematic analysis was conducted.

**Results:**

The analysis indicated two main themes: risk management and damage control in a restricted environment and compensating and reconstructing ordinariness—trying to make the best of it; each theme had three subthemes. The care environment seems to be experienced by staff as characterized by conflicting demands, thus constituting a gap between needs and what is possible to achieve—a balancing act that constitutes a constant struggle.

**Conclusions:**

The staff members’ constant struggle could be interpreted as conflicting moral and instrumental demands; they know what the youths need, but the environment of the secure youth homes demands the decorous behaviour of sociomaterial control practices—rather than care practices.

## Introduction

Providing care and treatment in the socially and spatially strictly regulated and limited environment of an institution can easily constitute a self-contradiction, potentially creating a range of challenges for the people inhabiting the environment: both enrolees and staff. Yet, locked care settings, such as institutions for young people, have existed throughout history—and still do to this day. Care environment research focuses on the importance of the surrounding environment for the individual—the space itself, its design, and materialities, as well as the subjective experiences and attributed meanings. This study is positioned within the broad and interdisciplinary field of care environment research and focuses on staff members’ experiences of a specific type of locked care environment: secure youth homes run by the Swedish state.

### The Swedish secure youth homes

In Sweden, according to law, youths up to 21 years old are placed at secure youth homes due to extensive care needs or sentences (Swedish Agency for Health Technology Assessment and Assessment of Social Services, 2016; The Care of Young Persons Act, 1990; The Law on Young Offenders, 1998). According to the Swedish National Board of Institutional Care, these secure youth homes aim “*to create better conditions for a socially functioning life without abuse and crime*” (The Swedish National Board of Institutional Care, 2021). These homes have several types of staff members, with the largest group being treatment assistants, who are responsible for providing everyday care in the wards and are thus spatially, socially, and emotionally closest to the youths (Andersson, 2021; Enell et al., 2018; Silow Kallenberg, 2016).

The secure youth homes constitute complex settings, where care and treatment intertwine with social protection and security issues in a care environment characterized by what Wästerfors called “*a tense everydayness*” 2019 (p. 12–13, authors' translation). This points to the contradictory traits of these homes—they constitute a homely everyday life while simultaneously being a strictly regulated, monotonous, and security-oriented environment (Biszczanik & Gruber, 2021; Wästerfors, 2019). These traits are made visible both through the spatial and material aspects of the care environment: through not only high fences, bullet-proof windows, security doors, and looking devices but also the rules and regulations shaping everyday life at secure youth homes, positioning them in between caring and guarding (Leviner et al., 2017; Sallnäs et al., 2017; Silow Kallenberg, 2016). This in-between position is further accentuated through the staff members’ legal mandate to use coercive means, such as isolation and restrictions of movement both within and outside the ward (The Care of Young Persons Act, 1990). In recent years, because of a government decision (Ministry of Social Affairs, 2020), there is an increased focus on security issues mainly through extended physical security arrangements and risk assessments throughout the organization responsible for the homes. Further, the Swedish secure youth homes could be argued as being in a unique position compared with the other Nordic countries, as well as European countries like the Netherlands, since it combines an already locked institutional environment with additional restrictive measures (Harder et al., 2013; Havre et al., 2018; Huhtanen et al., 2018).

The institutional care of youths has been unsatisfactory both from an international perspective and in relation to the Swedish secure youth homes—for example, in terms of remaining problems and relapse in criminality after discharge (Enell, 2015; Gudmundsdóttir & Nordqvist, 2007; Pettersson, 2010, 2017; Vinnerljung & Sallnäs, 2008; Vogel, 2012). Although institutional care may be the only solution under some circumstances, it can be insufficient or even harmful, especially if the placement lasts for extended periods and for more well-functioning youths (Andreassen, 2003; Dodge et al., 2006; Van Ryzin & Dishion, 2014). The unsatisfactory results are usually related to “contagious peer interactions”, meaning that young people with different types of destructive behaviours learn from each other when they are placed together in institutions (Bengtsson, 2012; Dodge et al., 2006; Van Ryzin & Dishion, 2014). That may partly be because involuntary institutional care and its sociospatial restrictions and regulations create a starting point where the young people build relationships with each other rather than with the staff (Nolbeck, 2022; Wästerfors, 2012).

### Research on institutional care environments

Although some studies on secure youth homes have touched on spaces and materialities as crucial parts of the experience of the care environment (see, for example Silow Kallenberg, 2016; Vogel, 2020; Wästerfors, 2019), few have focused on this. Biszczanik and Gruber stated that security work and security issues at secure youth homes implicate a “doing” involving the emotional work that the staff perform (Biszczanik & Gruber, 2021). Furthermore, secure youth homes constitute emotionally dense spaces in which emotions are tangible and constantly present in the interactions between youths and staff (see, for example Gradin Franzén, 2014; Silow Kallenberg, 2016; Wästerfors, 2019). As mentioned, the rules and routines and the design of the institutional environment create social distance between the staff and youths (Goffman, 1961; Nolbeck et al., 2020; Ugelvik et al., 2014). This social distancing is related to the youths’ interpretation of the spatial and material aspects of the care environment of these homes and the related rules (for example, the coercive means), as inscribed with the meaning of security, control, and lack of care (Nolbeck et al., 2020, 2022) and the staff members’ corresponding interpretation of the secure youth home as a dangerous place (Biszczanik & Gruber, 2021; Enell & Wilińska, 2021).

The care environment as a concept and phenomenon can thus be understood as a whole consisting of spatial, material, and social aspects. This understanding of the care environment relates to the concept of sociomateriality, which views spaces and material objects as intertwined with and inseparable from social interactions and practices (Orlikowski & Scott, 2008; T. R. Schatzki, 1991; Zieleniec, 2007). The interpretation of spaces and objects constitutes a part of one’s identity through individuals attributing to themselves the characteristics and symbols the environment conveys (Fransson et al., 2018; Østerberg, 1998). For example, a prison building with its fences and security arrangements has completely different connotations than a preschool building. Consequently, prisons and preschool buildings are interpreted in different ways, have different attached meanings, and followed by different internalized self-images of the people inhabiting these buildings (Jewkes, 2018; Ugelvik et al., 2014). Similarly, prisons and preschool buildings place different demands and expectations on the behavior of those on the inside (Goffman, 1956).

The concept of “at-homeness” as a metaphorical feeling of being at home (Saarnio et al., 2016, 2018, 2019) can be helpful to understand experiences of the intertwined social, spatial and material environment. Öhlén et al. reported that at-homeness constituted a contextually bound meaning and a continuum with the endpoints being metaphorically at home or homeless (Öhlén et al., 2014). Rather than an environment designed with the intention of being “homelike,” which could be understood as subjective, the sense of at-homeness and feelings of belonging are more important (Falk, 2010). However, objects and spaces with connotations to what is perceived as homelike, rather than institutional-like, can support and evoke a sense of at-homeness (Nolbeck et al., 2020, 2022).

Studies on the relationship between space and care have been performed in the context of mental and forensic psychiatric healthcare settings (Alexiou et al., 2016; Olausson et al., 2019; Ulrich et al., 2018; Wijk et al., 2019). The results revealed connections between the environment, stress, and aggression (Ulrich et al., 2018), as well as the importance of balance between private and social spheres (Evans, 2003; Olausson et al., 2021). They also highlighted how a purposefully designed environment affects lived experiences and identity by supporting the upholding of self and offering harmony and comfort (Olausson et al., 2021) as well as reorientation or a withholding of identity, where the environment either promotes a reorientation or “fits” with an already destructive self-image (James et al., 2021).

### Rationale and aim

Although the staff members are arguably the most important element of the care and treatment at secure youth homes, no study has examined their experiences of the institutional care environment or how it affects their work with the youths as well as their own work situation. Given the above outlined background and the fact that the care environment of Swedish secure youth homes is a relatively unexplored phenomenon, we aimed to examine staff members’ experiences of the institutional care environment in secure youth homes run by the Swedish National Board of Institutional Care. Investigating how spaces and objects are experienced by and affect the staff may reveal taken-for-granted features of interactions that affect the care and treatment work in secure youth homes (Latimer, 2018). The study contributes to expanding knowledge about what happens to the relationships and interactions between young people and staff in the spatially and materially restricted everyday life of Swedish secure youth homes and how the staff experience their work with the youths in this setting. Exploring the social, spatial, and material aspects of the care environment can increase the understanding of the staff members’ conditions for working with the young people in this context as well as the young people’s opportunities to develop and receive the support to which they are entitled.

## Methodology and methods

### Study design and participants

Given that the care environment of Swedish secure youth homes is relatively unexplored, we chose a qualitative design with data generated through focus group discussions (FGDs). The participants were staff members of two secure youth homes, and they were interviewed through FGDs in September and November 2020. The institutions were selected through purposeful sampling to achieve variety according to legal placement as well as the youths’ age and gender. This study is part of a larger interdisciplinary research project focusing on the physical environment of the secure youth homes. Within the project framework, the related information and request for participation were sent to all 21 secure youth homes in Sweden in 2017. Of them, 10 homes expressed interest in participating, of which two were included in the study: one housing boys up to the age of 16 years and one housing girls aged 14–20 years. The institutions represent both care, according to The Care of Young Persons Act, and sentences, according to The Law on Young Offenders (The Care of Young Persons Act, 1990; The Law on Young Offenders, 1998). Three FGDs were performed, comprising seven, four, and six staff member participants (total: 17; 6 men and 11 women). Two FGDs were performed at the institution housing boys, and one at the institution housing girls. The second scheduled FGD at the girls’ institution had to be cancelled due to the COVID-19 pandemic. The participants in the FGDs were treatment assistants (*n* = 13) and teachers (*n* = 4). The time spent working at their current secure youth home ranged from 2 to 25 years (mean: 7.2 years). Each FGD lasted 65–76 min and was audio recorded and transcribed verbatim.

Prior to data collection, two pilot FGDs were conducted with staff from two other institutions to test the questions and structure, leading to marginal adjustments of the questions. On the basis of the result of pilot FGDs, we also decided to introduce extracts from previously conducted interviews with youths at secure youth homes as facilitators to start the discussions. This decision was based on our previous research (Nolbeck et al., 2020, 2022) as well as the pilot FGDs showing that elements such as photos, sketches, or text can have a facilitative effect, enabling the participants to express their experiences and views about more abstract phenomena such as the care environment. The two interview extracts consisted of one page each of transcribed interview text that was chosen to present two different experiences of being cared for at a secure youth home. The first extract displayed a youth who describes the lock-in and the closed environment providing a sense of security and respite from a life with drug problems. The second extract displayed another youth expressing the longing to get out of the youth home. The interview extracts were read by the participants in the initial phase of the FGD.

### Focus group discussions

We chose to conduct FGDs because they are especially suitable when striving to access people’s knowledge, attitudes, or experiences and reveal group norms of a certain phenomenon considering what, how, and why the participants think the way they do (Kitzinger, 1994). Another reason is that we were primarily interested in the participants’ collective experiences, opinions, and thoughts about the care environment, rather than the experiences of the individuals. In FGDs, rather than individually interviewing the participants, the researcher takes on the role of facilitator of a group discussion focusing on a specific topic (Watts & Ebbutt, 1987), which captures both individual and collective experiences (Dahlin-Ivanoff & Holmgren, 2017). Considering that some people may have difficulty making themselves heard in a group, the facilitator of the discussion must be responsive and alert. In the present study, this was ensured by the presence of two researchers during each FGD: one who facilitated the group discussion and one who observed, took notes, and caught up on things that the facilitator missed out on (Watts & Ebbutt, 1987).

The FGDs started with a short presentation by each participant and the researchers, including name, profession, and, for the participants, how long they had worked in the field of youth care as well as at the current institution. The researchers informed the participants once again about the study aim. Thereafter, the participants read the transcribed interview extracts. After initial reflections on the interview extracts, the facilitator followed the discussion but avoided an interventionist role and instead awaited natural pauses in the conversation where an open-ended question could be interposed (Watts & Ebbutt, 1987). The open-ended questions used to facilitate the FGD are listed in Table I.

**Table I.** Questions used to facilitate the focus group discussions.

What are your reflections from reading the interview extracts? How do you think we are affected by spaces and objects we are surrounded by? What does the term ‘care environment’ mean to you? How do you experience the environment at your ward/in the classroom/at school? Do you feel that the environment affects your work with the youths, and in what way? Do you feel that the environment affects how the youths feel, and in what way? Does the environment affect your work environment, and in what way? In relation to your work with the youths, what opportunities do you see in/with the environment? In relation to your work with the youths, what obstacles do you see in/with the environment? What kind of care environment do you think is needed in the work with youths who stay in secure youth homes? Why is such environment needed?

All of these questions were addressed in the three FGDs.

The first author conducted the FGDs (facilitator role), with the second and last authors assuming the observer role in one and two FGDs, respectively.

### Ethical considerations and statement of rigour

Research in the context of involuntary institutional youth care places high demands on researchers due to ethical considerations and child rights perspectives (Källström & Andersson Bruck, 2017). In connection with previous studies at secure youth homes, an ethical codex was developed within the research project to analyse and prevent various ethical risks. In addition to children’s rights (United Nations Human rights Office of the High Commissioner, 1989), the ethical codex, which also formed the basis of the present study, is based on principles of non-maleficence, beneficence, autonomy, and justice (Beauchamp & Childress, 2013).

Prior to the FGDs and after obtaining consent from the head of the institutions, the staff were informed orally about the research project, its aim, and data collection method, through the managers passing on information from the researchers. The staff who wanted to take part in the study were given the date and time of the FGD by the researchers’ contact person at each institution (a treatment assistant in one case and a head of a ward in the other). While on-site, the staff members that showed up for the FGD were once again invited to the study by the researchers, who provided oral and written information at that time, including the opportunity to ask questions and time to consider the invitation while also stressing the voluntary nature of the study and their right to withdraw at any point. Finally, written consent was obtained from those who agreed to participate. Prior to the discussion, we emphasized the importance of confidentiality and respect for each other’s opinions, including that what is said in the room may not be passed on. The study was conducted ethically and responsibly, complying with all relevant legislation and declarations (Ministry of Education, 2003; The World Medical Association, 1964). All data were handled according to the General Data Protection Regulation (GDPR) (The European Parliament and the Council of the European Union, 2016). The research project also received approval from the Ethical Review Board (ID nr 1158–16, 2017-03-06).

None of the authors had any relation (work-related or other) to the participants included in the study. However, the first author has previous experience from both previous field work at the two included secure youth homes and with working in community-based and institutional youth care. All the authors have previous experience in conducting research within the current field. To ensure compliance with quality requirements for qualitative research, the COREQ 32-item checklist for interviews and focus groups (Tong et al., 2007) was used.

### Thematic analysis

The transcribed FGDs were analysed using thematic analysis according to Braun and Clarke (Braun & Clarke, 2006), which aims to search for, identify, and analyse patterns of meanings. Moreover, this type of analysis can be used with different theoretical frameworks and draw on different epistemological assumptions (Braun & Clarke, 2006). In this study, an inductive data-driven, primarily semantic thematic analysis was performed. In this case, “inductive” means that the data constituting the themes are strongly linked to each other rather than fit into an already existing theoretical framework (Braun & Clarke, 2006). Furthermore, here, “primarily semantic thematic analysis” means that the analysis focuses on the explicit, manifest meanings expressed by the participants, and does not attempt to further interpret their intentions, opinions, or values behind statements. However, such an analysis also involves interpretation and theorizing in relation to a broader context of meanings (Braun & Clarke, 2006).

Braun and Clarke outlined six steps when performing a thematic analysis (Braun & Clarke, 2006). However, the analysis process is characterized by constant movement from the whole of the data set to the parts and back to the whole, with writing as an integrated part of the analysis. In this sense, the analysis process is not linear (Braun & Clarke, 2006). The first step of the analysis involved familiarizing with the data by reading and rereading the whole data set while noting ideas and reflections. Second, initial codes were generated by identifying and labelling the features of the data relevant to the aim. Third, themes were searched for by collating codes and their associated data together into potential themes. Fourth, the themes were reviewed at two levels—in relation to the coded data extracts and the data set as a whole—and a thematic map was developed to obtain an overview of potential themes and subthemes. Fifth, the analysis proceeded by refining the themes and subthemes as well as the overall story they tell, including naming and defining each theme and subtheme. Finally, relevant and illustrative data extracts were selected and analysed. Here, additionally, the analysis was related back to the study aim, as well as the relevant literature and theory (Braun & Clarke, 2006).

## Findings

**Figure 1.**
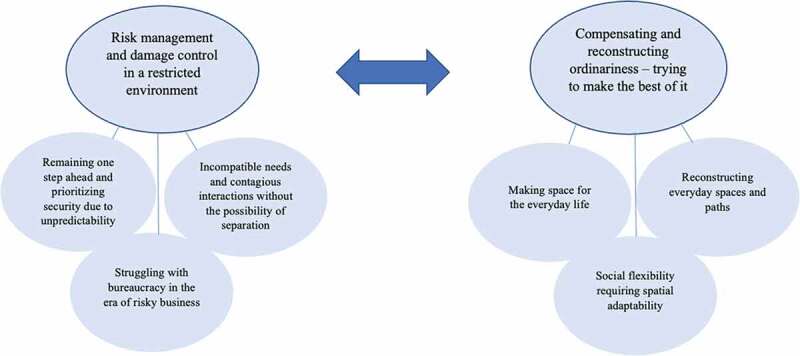
Thematic map of themes and subthemes.

The thematic analysis resulted in two main themes, which consisted of three subthemes each (Figure 1). The themes and subthemes are presented further below as headings and subheadings, along with their associated analysis. Extracts from the FGDs are presented in boxes, and shorter citations are highlighted using italics within the text. All the names used are fictitious to ensure anonymity.

The two main themes and subsequent subthemes are not placed in any hierarchical order; rather, they illustrate the tension and struggle the staff expressed as defining and characterizing their everyday work. In the data, it is evident that the staff are constantly balancing risk management and damage control while trying their best to compensate and reconstruct ordinariness. This balancing act seems like a constant struggle. Moreover, the environment of the secure youth homes seems to be experienced by the staff as one filled with conflicting demands and recurring situations that constitute a gap between what the staff believe is required and what is possible to achieve in the social and spatial environment. The main themes and subthemes and their subsequent analysis are further outlined below.

### Risk management and damage control in a restricted environment

The participants’ views on the care environment consist of material, spatial, and social aspects. Questions on security issues, damage control, and risk management are recurrent themes in the data. Specifically, the staff expressed how they are constantly trying to control and manage risks and potential dangers to minimize damage and contagious peer interactions. However, they struggle with both bureaucracy and unpredictability, as illustrated by the subthemes below.

#### Remaining one step ahead and prioritizing security due to unpredictability

The care environment is expressed as determined by—and thus changing with—a constantly changing youth collective at the ward, as illustrated by the following discussion:

*Viveca*: I would say that … yes, it’s [the care environment] here everywhere, but it moves, like, depending on where we are …

*Lisa*: Just like here at [secure youth home] there are different care environments in different wards …

*Viveca*: Yes, yes.

*Lisa*:…depending on what the clientele looks like.

Extract from FGD no. 3, the girls’ secure youth home.

Thus, the participants express it as a two-way process in which the care environment is dependent on the youths. The change in the care environment is thus often out of their hands, especially when a new youth is joining, as described by Ellen (FGD no. 3): “*So … (…) we never know (…) what, who will come [laughs]. What they bring with them*.” In this context, it becomes a preventive security measure to “*dress down*” the spatial and material environment, for the staff, other youths, and new youth themself. The experienced unpredictability of the youth collective also leads to the care environment being unpredictable and something that needs constant monitoring by the staff. Consequently, it becomes necessary to stay one step ahead and prioritize security. The staff expressed that for them to remove objects and lock doors, it is sufficient for one youth to be assessed as unable to cope with a more homely, everyday environment:

*Amalia*: And sometimes it does not go well at all. Of course, then you also must enforce the rules … that affect everyone, even those who have behaved very well and had a good time before. Then, you may have to start to … lock the kitchen and …

Extract from FGD no. 3, the girls’ secure youth home.

Specifically, the staff noted how they often must “*dress down*” a materially homely everyday environment to the security-related advantages of collective constraints, fewer objects, and locked doors. This unpredictability requires the staff to constantly “*stay one step ahead*”—preventive work that involves locking and unlocking doors, picking out and removing objects, as well as constantly evaluating the youth collective at the ward, and “*what they can handle*” at that very moment.

*Anna:* Then you must consider that when we work where we do and have the clientele we have, our opportunities are limited. (…) Eh, but then it’s based on the fact that security comes first, that they should not hurt themselves or anyone else, and that you get to sort of dress down. (…) you try to make it nicer. But then, there is always a security risk. Eh, and that one must consider. So, so that makes it a little difficult.

Extract from FGD no. 1, the boys’ secure youth home.

The contrast between risk management and damage control, as well as a more homely everyday environment, was highlighted throughout the FGDs. Here, to “*dress down*” was described as a quick and sudden process, whereas to “*dress up*” was described as a slower process, involving discussions and careful considerations among staff. In this context, Lisa (FGD no. 3) noted, “*You cannot put things out directly again but may make a deliberation and risk assessment*.” The staff also described how the youths, as part of their treatment, may “*work for*” some freedom, socially and spatially:

*Maria*: It is very individual, depending on the youth, but it is a matter of building trust. Partly that they should be able to trust us more, and we should be able to trust them more to avoid … yes, but escapes and relapses and so on.

Extract from FGD no. 3, the boys’ secure youth home.

Here, when the youth collective at the ward is assessed as “*ready*” and has the ability to “*handle*” a more homely everyday environment, there can be discussions and reconsiderations. However, security issues seem paramount, and this is motivated by several aspects:

*Jack*: So, security, it’s very important. For (…) … if a kid has locked himself in his room and found a weapon somewhere, because we do not have buildings that are proper, and maybe broken … Then, we should not go in. Not even the police go in, but they wait until the [policemen with] shields come in. So … we really have no protection, so we must always try to be prepared for such issues.

Extract from FGD no. 1, the boys’ secure youth home.

Thus, the youths’ ability to hurt themselves was another argument for “*dressing down*” the environment. Here, the glass trays in microwave ovens, coffee makers, fan glass, antenna sockets, tiles, windowsills, porcelain toilets, cosy lighting fixtures, and plants are described as potentially dangerous objects that they can either injure themselves with or use as weapons against staff. Amanda (FGD no. 2) concluded by saying, “*I always believe that security… (…) always comes first. And then you try to do as well as possible based on that*.”

#### Incompatible needs and contagious interactions without the possibility of separation

The participants expressed that the environment is both limited and limiting as it is crowded with too many youths and staff in a space that is too small. This has direct consequences, as illustrated by Maria (FGD no. 3): “*When we have the full number of girls, then there is no room around the kitchen table to all sit together and eat*.” Isaac (also FGD no. 3) agreed, saying, “*There is always someone who must pull an armchair to the dining table*.” Moreover, security issues are believed to be jeopardized, and crowdedness can lead to both staff and young people being easily irritated. The lack of more spacious rooms and fewer people per room is expressed as a problem—both at the wards and in schools.

However, the experience of small spaces with too many people seems to create the feeling that the ward or secure youth home is not only crowded in spatial terms but also socially and relationally. In this context, Ellen (FGD no. 3) stated, “*So, seven youths with various difficulties, it … it … it’s like having 30 sometimes*,” while Lisa (FGD no. 3) added, “*Actually, we need more staff. And then at the same time, we do not have space at the ward (…)*.”

*Viveca*: Yes, but it’s very crowded. Eh, the school and our ward are both very inefficiently soundproof. Eh … [sighs] yes.

*Ellen:* Yes, but there are small spaces and many youths. [general support] You notice when the wards have a reduction in placed youths, it’s fantastic (…) [general support] And then maybe it’s four instead of seven (youths). So, there is a very big difference.

Extract from FGD no. 3, the boys’ secure youth home.

The experiences of crowdedness and a socially dense environment seemed to be further accentuated through the expressed view that different groups of youths with varying needs are placed together. Expressions such as different youths are “*mixed wildly*” in the wards were recurrent in the data material. The different youths and needs were often considered incompatible and expressed in terms of something that needs to be “*balanced*” or “*set against each other*.” This was articulated as being further hindered by the cramped and restricted physical care environment. Consequently, the staff members often ended up using corrective measures towards all youths at the ward. This affects youths who “*behave*,” those who are “*calm*,” or the younger ones, who are then “*pushed away*” to their bedrooms because the staff do not want them to be “*dragged into*” conflicts or influenced by older youths or “*those who disturb*.”

Moreover, one experience associated with both the crowding and the “*mixture*” of different youths and needs was the view that there is a constantly imminent risk of contagious interactions. It was mainly expressed as between the “*calm*” youths or the younger ones who learn from the older criminals or those with other more difficult problems. “Calm” in this case refers to youths with less externalized behavioural problems, or younger youths with less extensive and more internalized problems. Here, the solution was expressed in terms of spatially separating different youths from each other. However, the restricted physical environment and crowded space of the ward, as well as the school spaces, makes it difficult or impossible to spatially separate them, as Amina explained:

*Amina*: So, we can have a ten-year-old with a sixteen-year-old, and this ten-year-old may have so many diagnoses and that there is so much neuropsychiatry involved. And this sixteen-year-old is a gang criminal, and they live in the same ward. And you do not want this ten-year-old to learn how to be … So, it’s very difficult.

Extract from focus group discussion, the boys’ secure youth home, FGD no. 2.

However, being unable to spatially separate the youths also has to do with the general organization of institutional care. The participants expressed wanting a more differentiated and target group-adapted organization, where placements are considered based on the youths’ needs as well as how the youth group at a ward functions. The present handling of placements, through a centralized decision-making process, was viewed as problematic in terms of the environmental prerequisites for care, as explained by Anna:

So, I would have liked to see more like in healthcare, where you (…) You’re on Thorax for help with your heart. I would have liked to see more of that in our organization. Like at our ward, we currently have five young people, three of whom are entitled to support due to disabilities, along with two criminals who are like in a gang war with each other. Eh, that’s difficult to work with, and it’s an environment that I do not think is fair to the youths. (…) I do not think it is a nice living environment for the children.

Extract from focus group discussion, the boys’ secure youth home, FGD no. 1.

Thus, the opportunity to spatially separate youths is expressed as a strong desire and solution to many problems that the staff encounter in their daily work; however, this is not easily achieved.

#### Struggling with bureaucracy in the era of risky business

Apart from the lack of spaces and perceived crowdedness, the participants noted that changes or renovations of the environment, such as repairing something or ordering new furniture, take “*too long*.” Thus, they expressed frustration over what they considered a bureaucratic and tortuous way to have changes made to their everyday environment:

*Jack*: Yes, I can think that one thing that is quite frustrating—everything takes so much time. (…) I do not understand why it takes so long because we are still a government authority. It should go at a much faster … faster pace. (…) We had to fix a fenced yard, well, the net there. It took six months to decide on the stitches, and then it took a year and a half to get … before we could have a fenced yard. It’s too long, so … and it affects the environment you work in and the security above all, which we must constantly talk about, security.

Extract from focus group discussion, the boys’ secure youth home, FGD no. 2.

The prolonged process of getting things renovated and restored affects the participants’ work with the youths, as described by Larry (FGD no. 1): “*But it affects like, the treatment (…) if we are just going to repaint a room or (…) something is broken, and (…) you have resolved the conflict (…) But all the time the reminder of it remains (…)*.” However, not only the treatment work but also the work environment for the staff is affected by the prolonged process, along with the lack of sufficient and “*approved*” physical spaces:

*Viveca*: For example, care in private at [ward], where there is only one bedroom and one bathroom (…)

*Amalia*: A bed. (…)

*Viveca*: If the staff is there, you basically sit on the floor. (…)

Extract from focus group discussion, the girls’ secure youth home, FGD no. 3.

Apart from the above, the staff noted that currently, there is much more of a focus on risk assessments and security issues—i.e., on the “*hard factors*” that are expressed as having direct consequences on the care environment. “Hard factors” in this context seem to imply a one-sided focus on risk assessments and physical security arrangements, at the expense of what participants rather refer to as “soft factors” that were more associated with a relational focus and more mundane and homelike objects and activities. Consequently, they hear a “*a lot of no*” on things that were previously considered okay or even part of the treatment—from social group activities to interior design and objects in the environment. The participants expressed an organizational view emphasizing assessments, documentation and evaluations and a general feeling that much more is nowadays considered “*dangerous*,”:

*Amanda: *But I know that when I worked at [department name] … we had a lot of lit candles, we had a fireplace … huge aquarium and … yes, but so on. All that has just disappeared. It gets … from a security point of view, there is no such thing left. (…)

*Facilitator*: What is it then that has … Why has it disappeared? So, what has changed?

*Amina*: So, I do not think there have been any more incidents because we never had an incident involving them, neither with the fire nor with those candles. It’s probably more about security issues. There are more risk assessments being made, and there is more focus on the hard (…) factors.

Extract from focus group discussion, the girls’ secure youth home, FGD no. 2.

Apart from the focus on security issues, the participants also described what they consider a “*different clientele*” today, with older youths with more mental health issues. However, they are unsure of whether the youths’ problems really are much more complex today or whether they are a consequence of changes in society, as Amina reflected: “*But it’s also a bit like what society looks like in general as well, I think. (…) So you want to put labels (…) …* .”

### Compensating and reconstructing ordinariness—trying to make the best of it

The staff constantly strives to support ordinariness and enable social skills training for the youths—treatment that requires everyday spaces, objects, and paths. However, the lack of this constantly annihilates and obstructs the participants’ work, as shown in the subthemes below.

#### Making space for everyday life

The participants expressed the importance of having spaces and material objects that enable youths to develop and refine social and practical skills, such as the “*basic skills you have in a family*.” This included everything from learning to wash cloths, putting dishes in the dish washer, cleaning, and interacting with others to doing so through constructive conflicts. Eva (FGD no. 3) said, “*I think above all [they need] a safe environment where you feel that (.) it is safe, (…) an open climate. So, I think not only the physical space but also the environment of the people around you*.” Similarly, many participants highlighted the need for a homelike, softer environment that is less institutional-like. Here, having access to common things you have in a home was emphasized as facilitating good relations between youths and staff, but also learning practical and social skills and preparing for life after the secure youth home:

*Ellen*: They do not have these routines with breakfast, lunch, dinner, and sleep at night and that, but you need to have these safe, stable routines, and the structure of a home environment (…) not so institutional. (…) As far as possible. With pillows on the couch and blankets and cosines and so on (…) and they get to be sad and angry and happy and [still have] goodnight hugs and all. (…) you just must prove that it is possible to trust adults. [general support]

Extract from focus group discussion, the girls’ secure youth home, FGD no. 3.

Moreover, everyday life includes the opportunity to choose for yourself. Having the opportunity to “*spruce up*” your own private room, be able to withdraw from social interaction, and spend time with yourself was emphasized as important in this context. However, a more homelike everyday life in these terms is often not possible due to security reasons and restrictions.

*Larry*: (…) When you do an activity, or you sit down, and everyone eats dinner, such basic simple things make them move forward in development in some way. And there it is lacking because it looks like it does, and we must accept the situation as well.

Extract from focus group discussion, the girls’ secure youth home, FGD no. 1.

#### Reconstructing everyday spaces and paths

The participants also expressed a desire to reconstruct everyday places and paths to the way they are in life outside secure youth homes. The idea that different activities require different spaces and that the change of activity is facilitated by the change of space or place is current in the data. Here, being able to walk on your own between different activities and places within the area of the secure youth home was expressed as promoting responsibility and motivation.

Moreover, the importance of creating different spaces for different activities within the institutional area where youths can develop practical skills as well as social skills through interaction was highlighted as an important part of the treatment. A good example seems to be the school, which is often in its own building. This sometimes enabled the youths to literally “*go to school*” and give at least a temporary feeling of openness and freedom, which is further promoted by letting youths from different wards go to school together. Dennis (FGD no. 2), who has worked for many years at the boys’ secure youth home, described what happened when they decided to move the school lessons out of the ward to its own building: “ *… and we then noticed a big difference that the students took the task seriously, that it was really like you put on your clothes, you take your … school materials, go down to school and then back to the ward*.” Amira (FGD no. 2) agreed and also described how the increased degrees of freedom through walking between different places within the institutional area is viewed as part of the treatment at her ward: “*And what we notice from our guys is that it is very much appreciated—this freedom. ‘We can move freely 50 metres, all by ourselves, without having any annoying staff behind our backs’*.”

The participants also expressed that the youths need natural situations to practice interaction with other youths and other people, both within the area of the secure youth home and in public environments—i.e., they need to be “*trained*” for life after the institution. In this context, the participants wished to “*get out*” more often with the youths to give them the opportunity to “*train*” in various social situations and that it was important to “*work them outward, (away) from here*,” as Viveca (FGD no. 3) expressed. However, because of the design of the environment, as well as the organizational focus on security issues and risk assessments, this was articulated as being difficult to realize. Additionally, Eva, a schoolteacher, described a wish for an environment that supports the youths’ ability to be trained in social interactions as well as responsibility, as in an ordinary school (FGD no. 3):

*Eva:* (…) So, I would have liked … yes, but preferably a common schoolhouse where we … yes, have … (…) yes, but open spaces where they might actually be allowed to have a locker … be responsible for their own things. [general support] Eh, those who can handle it. Eh, and give them a chance.

Extract from focus group discussion, the girls’ secure youth home, FGD no. 3.

However, the opportunity to increase degrees of freedom, such as walking freely between activities and spaces, is something that needs to be earned or used as “*reinforcement*,” or reward for socially acceptable behaviour. The youths are said to need to show “*that they are capable of*” dealing with increased freedom. Thus, walking freely from one building to another becomes a responsibility that is earned and a step forward in the treatment and development process.

#### Social flexibility requiring spatial adaptability

The participants emphasized that their everyday life is characterized by constant flexibility and adaptation. Specifically, they try to make adaptations to the needs of each youth in a collective and limited institutional environment. In other words, they do the best they can and try to make the best of the situation. However, social flexibility does not seem to be accompanied by spatial or material flexibility. The participants described how their everyday life consists of contradictions, such as demands to work individually with the youths while simultaneously being in an environment with security rules and organizational challenges that limit that possibility. In this context, Larry (FGD no. 1) noted, “*I think it’s always a conflict with eh, the coziness factor or how nice and good it should be, with security issues. Just as it is with us having the same rules (for all youths), but we must work individually”.*

The ability to adapt the environment to individual needs and to the conditions of the youth collective was repeated throughout the data. The participants expressed that the “*dream*” would be to have an environment where you can change the setting as on a theatre stage, depending on youths’ needs. The wish for a flexible environment was argued for both at the ward and in the school spaces and expressed in terms of getting spatial and material support for the care and treatment work that they are required to perform with the youths, both individually and collectively. The lack of spatial flexibility had consequences for both care and treatment and the work environment:

*Facilitator*: So (…) but what consequences does it have for the work with the young people and for you …

*Michaela*: That we wear ourselves out.

*Facilitator*: Yes, your work environment.

*Michaela:* Yes [laughs].

*Larry:* Yes, partly the energy there, but there is also an alignment sometimes on the care or on the treatment. That we do it a certain way because that’s what works here.

Extract from focus group discussion, the girls’ secure youth home, FGD no. 1.

Visions such as having two mirror-inverted identical wards or two kitchens—one open and one with the possibility of delimiting into several smaller spaces—were also emphasized. Larry summarized this as follows: “(…) *we have a complex business, and then we get to have a complex premise as well*.”

## Discussion

Both the United Nations Child Rights Committee and the Ombudsman for Children in Sweden have repeatedly criticized Sweden for its secure youth homes—a critique that has involved spatial and material environmental aspects, such as restrictions on movement and isolation (Lööf, 2011; Ombudsman for Children, 2019; United Nations Committee on the Rights of the Child, 2015).

Our findings indicated that staff members constantly struggle with risk management and damage control, yet are still trying to compensate for and reconstruct ordinariness, both in terms of social interactions and through the restricted spatial and material environment. Their aspirations have direct spatial and material dimensions in that they involve a constant activity in the form of locking and unlocking doors, retrieving, and removing material objects, and being constantly on guard. These are actions similar to what Mol, Moser, and Pols called “*tinkering*”, which involves constant doing, evaluating, and fine-tuning in care practices (Mol, Moser, Piras, et al., 2010; Mol, Moser, & Pols, 2010). Notably, the practices within secure youth homes do not necessarily constitute care practices, as shown in previous studies (Nolbeck et al., 2022). Our studies have demonstrated that the staff are in control of the environment/settings and objects and, thus, the definition of situations. However, these findings are understood in a different light from the results of the present study. Not only do the youths experience the environment as distance making and inscribed with security but also do staff members struggle with this and with the contradictory requirements of simultaneously having to create the feeling of everyday life at the ward and upholding measures related to security and control.

The constant “*tinkering*” and social adaptation that the staff members express as necessary could be related to what Biszczanik and Gruber called “*being on guard without showing it*” (p. 58). This relates to the staff constantly observing and assessing the youths, both as individuals and as a collective, without trying to make it visible (Biszczanik & Gruber, 2021). However, our data indicated that being on guard also constitutes practices involving locking and unlocking doors and removing potentially dangerous objects, thereby also shaping and reshaping spaces. These sociomaterial control practices are visible and acknowledged by the youths, as they are intertwined with spaces and material objects (Orlikowski & Scott, 2008; T. R. Schatzki, 1991; T. Schatzki, 2019; T. R. Schatzki et al., 2018; Zieleniec 2007).

The staff members’ striving for a more homely environment can be related to the concept of “at-homeness” conceptualized by Öhlén et. al. as a contextualized continuum between metaphorically feeling at home and homeless (Öhlén et al., 2014). In line with previous studies (Nolbeck et al., 2020, 2022), our findings indicate that material objects and spaces with associations to a homelike, rather than an institutional-like, environment are perceived by staff as supporting a sense of at-homeness. However, neither the creation of an environment intended to be homelike in terms of spatial design nor creating conditions for a sense of “at-homeness” was easily achieved within the secure youth homes. More specifically, everyday life is challenged by the strictly regulated and security-oriented environment that creates an everydayness that is “tense” (Wästerfors, 2019) (pp. 12–13). This tenseness is related to the experience of youths with contradicting needs being placed together and to the experience of an increased organizational emphasis on “security work” displaying shifting interpretations of the concept of “security” among the staff. Although some participants emphasized the importance of relationships, i.e., getting to know the youths and providing a softer and safer environment, others focused on physical security arrangements and the “protection” of staff or “other youths”. The expressions related to the concept of security can thus be interpreted as situated on a scale: from safety-creating care through control and collective strategies to collective repressiveness.

The findings also indicate that the staff members wish for objects and access to spaces that can support them in their creation of a more homely, everyday environment and evoking a sense of “at-homeness” with the youths. This is also what is considered important to support care and prosocial change. However, as Biszczanik and Gruber stated, security-inscribed settings signal a sense of danger, which creates feelings of uncertainty and insecurity with the staff (Biszczanik & Gruber, 2021). This is evident in expressions on security as “*always coming first*” and also in reflections on the development of institutional care towards risk orientation and security, which have more of a focus on “*on hard factors*” and “*a lot of no*.”

The various interpretations of the concept of security can thus also be related to staff members’ individual understanding of security at the secure youth home. Additionally, to what way the organizational focus on “security work” as decided by the Swedish government (Ministry of Social Affairs, 2020) is translated and implemented in the organization and, thus, how it is interpreted and implemented by the staff. The participants’ expressions related to security issues also bear connotations to the discourses of “child in danger” and “dangerous child” (Donzelot, 1997) in their wishes to spatially separate different youths from each other and their striving to not let the “*calm ones*” or the younger ones be harmed by the “*ones that disturb*” or the older, criminal youths. Here, the emphasis on security within the data could easily point to the domination of the discourse on the “*dangerous child*.” Specifically, the talk about “*dressing down*” the homelike environment and the constant assessment of what the youths, both individually and as a collective, can “*handle*” as well as the conditioning of increased degrees of freedom could be viewed as examples of this and has been confirmed in previous studies (see, for example (Silow Kallenberg, 2016)).

However, without the spatial and material flexibility supporting their work, the staff end up constantly balancing sociomaterial control practices promoted through the organizational emphasis on security made visible in the spatial environment—and compensating for and constructing ordinariness. The latter constitutes what they perceive as a basis for care and treatment. Thus, they try their best and wear themselves out, as expressed by Larry and Michaela. At the same time, the spatial and material environment signals danger and risk management, which, per Goffman, could be interpreted as an “*instrumental decorum*” (p. 107) that the staff needs to conform to (Goffman, 1956). A decorum recently and from the staff members’ expressions was emphasized through the government-initiated focus on security issues. Goffman conceptualized decorous behaviour as both moral (such as respect for another person’s integrity) and instrumental demands, which refer to requirements enacted by the employer (Goffman, 1956 p. 107). Additionally, Goffman stated that decorous behaviour may implicate showing respect for the setting one finds oneself in, and that this “*may (…) be motivated by a desire to impress the audience favourably or avoid sanctions*” (Goffman, 1956 pp. 108–109). The staff members’ constant struggle between risk management and control, on the one hand, and striving to create conditions for a more everyday environment, on the other, could be interpreted as conflicting moral and instrumental demands. In other words, they know what the youths need, but the environment of the secure youth homes and the organizational emphasis on security, physical security arrangements and risk assessments demand the decorous behaviour of sociomaterial control practices rather than caring practices.

The control over settings and objects is not in the hands of the staff, as indicated by our findings. Our results thus present a more nuanced picture by providing an understanding of the staff members’ prerequisites and challenges in creating a functional and caring environment. In this context, in the gap between what the staff understands that the youth need and what the care environment supports, there remain strivings and wishes, which cause staff to try to create a metaphorical space of the sense of at-homeness rather than homelessness (Öhlén et al., 2014)—i.e., a space for closeness, familiarity, comfort, harmony, and integrity (Olausson et al., 2021; Saarnio et al., 2019). However, these strivings usually remain merely strivings, because the care environment rather hinders both a homelike physical environment and social practices that could enable a feeling of at-homeness—rather than support them.

## Conclusion

In conclusion, staff members have some different interpretations of the concept of security, how it should be implemented, and how it should relate to a more homelike environment in social, spatial, and material terms. The staff found that the environment is shaped by, and changes with, those who inhabit it: they quickly step down from a homely environment when necessary but apply extensive risk assessments and considerations before stepping up again. This indicates that the environment is not static but emphasizes the importance of considering spaces and materialities as intertwined with social practices, as offered by the sociomateriality perspective. Furthermore, the institutions’ sociomateriality gives rise to rational, but not necessarily caring, practices—for example, the idea that access to certain spaces and objects must be earned by the youths or to use collective constraints. Finally, organizational, managerial, and governance aspects in relation to care environmental issues need to be studied in more detail in future research.

This study contributes to expanding knowledge on care environments as consisting of intertwined social, spatial, and material aspects in line with the theoretical concept of sociomateriality. It points to the importance of, within the organization, discussing how sociomaterial concepts as “security” and “homelike” should be interpreted, made visible and made practice in everyday life. This is to contribute to an environment with an emphasis on caring and a sense of safety for both youths and staff.

## Strengths and limitations

A major study strength is FGDs, which enable participants to interact in a discussion considering a specific topic of interest. An alternative data collection method could have been in-depth interviews with individual staff members. However, as the interest of this study was in the staff members’ experiences of the care environment, it can be said that the dynamics of the focus group method facilitated the participants in verbalizing their experiences on this complex phenomenon. Furthermore, the method also allowed for expressions that would not have come to the surface in one-to-one interview sessions and is especially suitable when using open-ended questions (Kitzinger, 1994).

Furthermore, the FGDs in the present study followed the recommendations of including 4–8 participants per FGD (Dahlin-Ivanoff & Holmgren, 2017; Kitzinger, 1994). Before the group discussion began, the researchers emphasized the importance of confidentiality and respect for each other’s opinions. This likely made the participants feel safe during the discussions. All participants in the respective groups also knew each other because they worked at the same institution. However, this can be both a disadvantage and an advantage. In this case, this can be seen as an advantage, as the dynamics of the group discussions were characterized by an open accepting climate. Furthermore, the participants in the FGDs were from different departments, which contributed to the open climate because, rather than guarding themselves in front of a co-worker, they could openly discuss any similarities and differences. At the same time, different opinions arose during the discussions, which can further be seen as a strength in that the participants dared to express different views.

Although the interview extracts may have at least partly influenced the participants, we have seen in our previous studies that this type of facilitating material supports the participants in expressing opinions and experiences about such an abstract phenomenon as the care environment. Initially asking participants for their general reflections on the interview extracts thus naturally opened the discussion, engaging them in the collective activity of reflecting on what they had read (Kitzinger, 1994). However, there is a risk that this study’s sample of institutions as well as participants was biased as the authors had to rely on gatekeepers to grant them access, possibly leading to a sample of participants with an initial interest in and experience of reflecting on care environment issues, as well as those highlighting only what they consider favourable. However, the participants not only expressed positive experiences but also seemed to wish for changes.

One of the researchers who conducted the data collection had previous experience of working with youths in social out-of-home care, and specifically on a secure youth home. This can pose both a challenge and an advantage in relation to preunderstanding. However, the other researchers who undertook the observer role during the FGDs had no such experience. There is always a risk, in qualitative research, as in all research, of over-interpretation, bias, and assumptions that affect the findings. However, given that not only the researcher with experience working at a secure youth home conducted the data but also each author assumed different roles and continuously discussed, critically analysed, and revised the findings, it can be assumed that any such impact has been reduced.

## Appendix

Tong, A, Sainsbury, P, Craig, J. Consolidated criteria for reporting qualitative research (COREQ): a 32-item checklist for interviews and focus groups. IJQHC; 2007;19(6):349–57. https://doi-org.ezproxy.ub.gu.se/10.1093/intqhc/mzm042

**Table:**

No Item	Guide questions/description	Compliance during research process
*Domain 1: Research team and reflexivity* Personal Characteristics		
1. Interviewer/facilitator	Which author/s conducted the interview or focus group?	The first author conducted the focus group discussions (FGDs) (facilitator role) with the second author (observer role, one FGD) and the last author (observer role, two FGDs).
2. Credentials	What were the researcher’s credentials? e.g., PhD, MD	The first author is a Ph.D. in Health and Care Sciences, a Master of Medicine with a major in Public Health and a post doctoral researcher in Social Work. The second author is RN, Ph.D. in nursing. The third author is a professor in Architecture and Civil Engineering. The fourth author is a Ph.D. in Architecture and a lecturer in Criminology, within the field of Police Work. The last author is an RN, professor of nursing.
3. Occupation	What was their occupation at the time of the study?	The first author was employed as a Ph.D. student at the Institute of Health and Care Sciences, University of Gothenburg, Sweden, at the time of the study. The second author is employed as a Ph.D. in nursing at the University of Gothenburg and Sahlgrenska University Hospital, Sweden. The third author is employed as a professor in the Department of Architecture and Civil Engineering, Chalmers University of Technology, and is the director at the Centre for Healthcare Architecture, Gothenburg, Sweden. The fourth author is employed as a Ph.D. in Architecture and a lecturer in Criminology, Faculty of Police Work, University of Borås. The last author is employed as a professor of nursing at the University of Gothenburg and Sahlgrenska University hospital. She is also a visiting professor at the Centre for Healthcare Architecture, Chalmers Architecture and Civil Engineering, Gothenburg.
4. Gender	Was the researcher male or female?	Four of the researchers are women, and one is male.
5. Experience and training	What experience or training did the researcher have?	All researchers have previous experience in the research field. The first author conducted this study at the end of her Ph.D. training and had conducted three previous studies under supervision. The other researchers have extensive knowledge and training, both in different research fields and methodological approaches.
Relationship with participants		
6. Relationship established	Was a relationship established prior to study commencement?	None of the authors had any relation (work-related or other) to the participants, prior to the study. However, the study is part of a larger interdisciplinary research project, focusing on the physical environment of the special youth homes. Within the framework of this project, information and the request for participation was sent to all special youth homes in Sweden in 2017. Of the total 21 homes, 10 showed interest in participating. From these, the sample of the current study was drawn. This means that the first and second authors have made previous visits to the two special youth homes included in the current study. However, they did not know the participants prior to the study.
7. Participant knowledge of the interviewer	What did the participants know about the researcher? e.g., personal goals, reasons for doing the research	The participants knew/got to know the purpose of the research project as well as the larger project, within which several studies have been done. They were told the names of the researchers and were informed briefly about their background, employment, and title. Regarding private opinions and values, the researchers did not share any of them because it could have affected the participants, and the research project negatively.
8. Interviewer characteristics	What characteristics were reported about the interviewer/facilitator? e.g., Bias, assumptions, reasons and interests in the research topic	In the manuscript, it is stated that the first author has previous experience from both previous field work at the two included special youth homes and working in community-based and institutional youth care. All the authors have previous experience in conducting research within the field current field. Further, in the Strengths and Limitations section, we address the issues of potential bias, assumptions, reasons, and interests in the research topic.
*Domain 2: study design* Theoretical framework		
9. Methodological orientation and Theory	What methodological orientation was stated to underpin the study? e.g., grounded theory, discourse analysis, ethnography, phenomenology, content analysis	The study was conducted using a qualitative approach, with data collected through FGDs. The study draws on the concept of sociomateriality (Orlikowski and Scott, 2008), and Goffman’s dramaturgical perspective (1956, 1961). However, the interpretation of the findings is primarily on a semantic, manifest level, focusing on the explicit statements of the participants (not trying to further interpret the intentions, opinions, or values behind a statement).
Participant selection 10. Sampling	How were participants selected? e.g., purposive, convenience, consecutive, snowball	The institutions were selected through purposeful sampling, aiming to achieve variety according to legal placement as well as age and gender of the youths. The study is part of a larger interdisciplinary research project, focusing on the physical environment of the special youth homes. Within the framework of this project, information and the request for participation was sent to all special youth homes in Sweden in 2017. Of the total 21 special youth homes, 10 showed interest in participating. From these, the sample of the current study was drawn.
11. Method of approach	How were participants approached? e.g., face-to-face, telephone, mail, email	Prior to the FGDs, and after obtaining consent from the head of the institutions, the staff were informed orally, through the managers passing on information from the researchers. The staff who wanted to take part in the study were given the date and time of the FGD by the researchers’ contact person at each institution (a treatment assistant in one case, and a head of a ward in the other). While on-site, the staff members that showed up for the FGDs were once again invited to the study by the researchers, who provided oral and written information as well as the opportunity to ask questions and time to consider the invitation, while stressing the voluntary nature of the study and their right to withdraw at any point. Finally, written consent was obtained for those who agreed to participate.
12. Sample size	How many participants were in the study?	In total, 17 staff members took part in the study. The three FGDs comprised of 7, 4, and 6 participants respectively.
13. Non-participation	How many people refused to participate or dropped out? Reasons?	All participants who announced in advance that they wanted to participate did so, except for one who could not leave the ward. The reason for this was understaffing.
Setting 14. Setting of data collection	Where was the data collected? e.g., home, clinic, workplace	The data was collected and the FGDs were performed in the special youth homes. They were not conducted in a ward but in a nearby empty building in one case (the boys’ special youth home) and in a conference room in an administrative building in the other (the girls’ special youth home).
15. Presence of non-participants	Was anyone else present besides the participants and researchers?	No
16. Description of sample	What are the important characteristics of the sample? e.g., demographic data, date	The participants consisted of six men and 11 women, of which 13 were care assistants and four were teachers. The time spent working at the current special youth home varied between 2 and 25 years (mean was 7.2).
Data collection 17. Interview guide	Were questions, prompts, guides provided by the authors? Was it pilot tested?	Two interview extracts were read by the participants in the initial phase of the FGDs. The extracts were used as facilitators to start the discussions. In addition, open-ended questions were used to facilitate the group discussion (see Table I,). Moreover, prior to the data collection, two pilot FGDs were conducted with staff from two other institutions to test the questions and structure, leading to marginal adjustments of the questions. This was also done to introduce the interview extracts from previously conducted interviews, as described above.
18. Repeat interviews	Were repeat interviews carried out? If yes, how many?	No.
19. Audio/visual recording	Did the research use audio or visual recording to collect the data?	The FGDs were audio recorded.
20. Field notes	Were field notes made during and/or after the interview or focus group?	No, this study was not ethnographic but qualitative and used focus groups as data collection method.
21. Duration	What was the duration of the interviews or focus group?	Each focus group took between 65 and 76 minutes
22. Data saturation	Was data saturation discussed?	It was established early that the material from the focus groups was very rich. Because the research object touches on such a limited and specific area as the care environment in the special youth homes, saturation was achieved more easily than if the study had touched on more general experiences.
23. Transcripts returned	Were transcripts returned to participants for comment and/or correction?	No. However, we emphasized that the participants were welcome to contact us afterwards.
*Domain 3: analysis and findings* Data analysis		
24. Number of data coders	How many data coders coded the data?	The first author did the initial coding, and thereafter worked in close cooperation with the last author to review the codes. Thereafter, the codes, subthemes, and themes were reviewed by the second author, and the subthemes and themes were reviewed of all the authors.
25. Description of the coding tree	Did authors provide a description of the coding tree?	The process of thematic analysis by Braun and Clarke (2006) is described in the methods section.
26. Derivation of themes	Were themes identified in advance or derived from the data?	The subthemes and themes were derived from the data, and hence grounded in empiricism.
27. Software	What software, if applicable, was used to manage the data?	The data was managed using an ordinary word processor program.
28. Participant checking	Did participants provide feedback on the findings?	No
Reporting 29. Quotations presented	Were participant quotations presented to illustrate the themes/findings? Was each quotation identified? e.g., participant number	Yes, quotations were used to illustrate findings, and fictious participant names were used to ensure anonymity. Yes, each quotation was identified in the interview transcripts.
30. Data and findings consistent	Was there consistency between the data presented and the findings?	The use of thematic analysis helped us to clarify the correspondence between the analysis, findings, and discussion.
31. Clarity of major themes	Were major themes clearly presented in the findings?	We have used several simultaneous ways to present our findings in as clear a way as possible to the reader. We have both inserted figures on the themes and sub-themes and used headings that categorize them. In addition, we have written introductions to each theme.
32. Clarity of minor themes	Is there a description of diverse cases or discussion of minor themes?	The themes and sub-themes that were identified were consistent throughout all FGDs and in the data material as a whole. Any disagreements mainly concerned the development of the special youth homes over time, where some participants had worked longer and thus had more extensive experience. We report on this in the manuscript.
